# Reviews

**Published:** 1884-01

**Authors:** 


### REVIEWS.

HORSES' TEETH: A TREATISE ON THEIR MODE OF DEVELOPMENT, ANATOMY, MICROSCOPY, PATHOLOGY AND DENTISTRY; COMPARED WITH THE TEETH OF MANY OTHER LAND AND MARINE ANIMALS, BOTH LIVING AND EXTINCT; WITH A VOCABULARY AND COPIOUS EXTRACTS FROM THE WORKS OF ODONTOLOGISTS AND VETERINARIANS. By William H. Clarke. Second Edition Revised. New York, W. R. Jenkins. 1884.

The Journal for January, 1881, contains a review of the first edition of this work, commending it in high terms of approbation. That edition has been so favorably received that the second one makes its appearance as a natural corollary. In it some improvements are to be noted in the shape of an Appendix, numerous illustrations, a new Index and much additional matter to the Text and Vocabulary. Many errors to be found in the first edition are here corrected and the book as it now stands is all that could be desired on the subject it treats of. As was remarked in a former review: "The work contains much besides the matter pertaining to horses' teeth, the teeth of many other animals being described and compared with those of the horse: in fact the work might be very properly entitled "Teeth" instead of "Horses' Teeth." It gives the history of the evolution of the horse from early geological periods, the half-teeth, which the author has named *Remnant Teeth*, being traced back to the Eocene period, at which time they were functionally developed. This fact throws light on what has been a mystery, and the author appears to have made a discovery.

We repeat our former praises of this work and hope it will continue its successful career of usefulness. C.

THE TREATMENT OF WOUNDS AS BASED ON EVOLUTIONARY LAWS. By C. Pitfield Mitchell, Member of the Royal College of Surgeons, etc. New York, J. H. Vail & Co. 1883.

The law of Evolution or Darwinism, as it is popularly styled, influences powerfully the endeavors of scientific men in every field of investigation. Cosmology, philosophy, physiology, ethics, etc. are all affected by this theory, and Dr. Mitchell, in the above treatise brings it to bear in surgery, on an exceedingly important and much discussed subject.

The matter is opportune as the universally accepted "antiseptic method" of Prof. Lister is gradually proving itself a failure, inasmuch as it interferes with the healing process in wounds, not only by bringing in contact with the tissues, chemical agents which tend to disturb the reparative action, but also by excluding an element best calculated to secure direct union, viz:—atmospheric air.

The process, according to the author, is a purely physiological one in the wounds referred to in his article, namely, those surgically-inflicted in a patient whose state and surrounding conditions are normal, or as he expresses it, "the *outer* and *inner* environments being natural."*

* It may not be out of place here to refer to an article by Mr. Kitchen, in No. 98 of the Veterinary Journal, which he especially recommends that free access of atmospheric air be allowed in the treatment of quitter wounds.

"In such wounds the *disentegration* of tissue is directly caused by the force which divides the histological particles, and ceases with the closure of vessels. With the glazing of the exudative plasma, the wound being open to the atmosphere, the *integrative* stage begins, and if the surfaces lie in apposition, union results without further functional or structural disorder." It also conforms to the law of evolution, "beginning with an effusion of simple, undifferentiated, generalized plasma, there are developed in different cells, and finally relatively complex and specialized vascular, granulation, and connective tissues and functions and structures are reintegrated." "Without functional harmony," he says, "between the exposed structures and the medium in contact with them, the functional and structural variations which constitute reparative processes could never have become established. While the air was competent to break down tissues unprotected by the skin, the evolution of constructive changes was manifestly impossible."

Natural selection has its influence. "Animals whose wounds were not repaired would strive in the struggle for existence under a heavy disability. The non-union of muscles torn and limbs broken during the encounters with prey and enemies would lead to incapacity for further encounters, and a dying off without issue; while others whose injuries were repaired would escape the direct hinderances and indirect dangers to life entailed by unhealed wounds and would reproduce their kind. Therefore by selective action there must ultimately result a type of blood and tissue capable of withstanding the impact of ordinary atmospheric forces."

In the domain of facts, Dr. Mitchell finds ample testimony in the data furnished by Dr. Borland and Mr. Lawson Tait who treated their wounds, in "wilful disregard" to Prof. Lister's doctrine, by permitting free contact with the atmospheric air.

Of course the Doctor does not reject antiseptic methods *in toto.* They are indispensable when the process ceases to be physiological and becomes pathological, the germicidal properties of the spray being indicated in the latter but not in the former condition. He considers their success as due in many cases to the great cleanliness observed.

In surgical operations the writer recommends that the instrument, the hands of the operater and assistants and the surface to be operated on be thoroughly cleansed and dried. Old, washed and very dry linen should be used instead of sponges or absorbent cotton. The exuded plasma should be protected and permitted to form a pellicle for the surfaces by being exposed to the air until dry. Torsion is to be substituted for the ligature. Metallic sutures are to be applied without straining or constricting the tissues. The cutaneous surface is to be washed and dried and *special care taken not to allow the cleansing materials to touch the flap borders.* The wound is now covered with a pad of absorbent cotten enveloped in two or three layers of washed linen of fine texture, to be renewed at the end of a week.

The work is to be commended as showing that in many cases simple measures are superior to complicated ones and as tending to bring into harmony the truths of surgery with the laws of nature.

Every Veterinary Surgeon should procure a copy and make the subject a matter of special study. J. O'L.

AIDE—MÉMOIRE DU VÉTÉRINAIRE MEDECINE, CHIRURGIE, OBSTETRIQUE, FORMULES, POLICE SANITAIRE ET JURISPRUDENCE COMMERCIAL. Par Jules Signol, Vétérinaire a Paris, Membre de la Sociétié Central de Medecine Vétérinaire de Paris, etc. Paris, J. B. Bailliere et fils, 1884. For sale by W. R. Jenkins, 850 Sixth Avenue, New York.

This concise and practical "Vade Mecum" contains condensed as in a nutshell, a summary of everything that a Veterinarian should know in Veterinary Medicine, Surgery, Obstetrics, Therapeutics, Public Hygiene and Jurisprudence.

The work is profusely illustrated by fine engravings, and the author has sought his information in the writings of such well known men as Pasteur, Colin, Zundel, etc.

The book is, without doubt, a very useful one and we hope to see it translated before long into the English language.

THE PHYSICIAN'S VISITING LIST FOR 1884. Blakiston, Son & Co., Philadelphia.

This very popular Visiting List makes its appearance this year with interesting additional features, viz:—Sylvester's method for producing artificial respiration and a diagram of the chest as an aid in diagnosis, stronger covers and a new pencil of special manufacture.

Veterinary Surgeons would find it very convenient in their practice.

*IN PREPARATION.*—THE RELATION OF ANIMAL DISEASES TO THE PUBLIC HEALTH, and their Prevention, with a Brief Historical Sketch of the Development of Veterinary Medicine, from the Earliest Ages to the Present Time; and a Critical Historical Sketch of the Leading Schools of the World, showing the Reasons which led to their Foundation, and with the endeavor to draw from their Experiences Teachings of value toward the Establishment of a General Veterinary Police-hygienic System and Veterinary Schools in this Country. By Frank S. Billings, Veterinary Surgeon, Graduate of the Royal Veterinary Institute, Berlin; Member of the Royal Veterinary Association of the Province of Brandenburg, Prussia; Honorary Member of the Veterinary Society of Montreal, Canada, etc., etc. 1 vol., 8vo.

The readers of the Journal will be pleased to learn that the above named book will soon leave the hands of the printer and will no doubt hasten to avail themselves of such a favorable opportunity of obtaining information on the interesting subject therein contained, the name of the author being a sufficient guarantee of the high character of the work. C.

